# Systemic Lupus Erythematosus Research: A Bibliometric Analysis over a 50-Year Period

**DOI:** 10.3390/ijerph18137095

**Published:** 2021-07-02

**Authors:** Malcolm Koo

**Affiliations:** 1 Graduate Institute of Long-term Care, Tzu Chi University of Science and Technology, Hualien City, Hualien 970046, Taiwan; m.koo@utoronto.ca; Tel.: +886-3-857-2158 (ext. 2206); 2 Dalla Lana School of Public Health, University of Toronto, Toronto, ON M5T 3M7, Canada

**Keywords:** systemic lupus erythematosus, bibliometric analysis, network analysis, research hotspots, research trends, bibliometrix

## Abstract

Bibliometric analysis is a well-established approach to quantitatively assess scholarly productivity. However, there have been few assessments of research productivity on systemic lupus erythematosus (SLE) to date. The aim of this study was to analyze global research productivity through original articles published in journals indexed by the Web of Science from 1971 to 2020. Bibliometric data was obtained from the Science Citation Index Expanded in the Web of Science Core Collection database. Only original articles published between 1971 and 2020 on SLE were included in the analysis. Over the 50-year period, publication production in SLE research has steadily increased with a mean annual growth rate of 8.0%. A total of 44,967 articles published in 3435 different journals were identified. The journal *Lupus* published the largest number of articles (n = 3371; 8.0%). A total of 148 countries and regions contributed to the articles. The global productivity ranking was led by the United States (n = 11,244, 25.0%), followed by China (n = 4893, 10.9%). A three-field plot showed that the Oklahoma Medical Research Foundation and the Johns Hopkins University together contributed 18.5% of all articles from the United States. A co-occurrence network analysis revealed five highly connected clusters of SLE research. In conclusion, this bibliometric analysis provided a comprehensive overview of the status of SLE research, which could enable a better understanding of the development in this field in the past 50 years.

## 1. Introduction

Systemic lupus erythematosus (SLE) is a chronic autoimmune disease characterized by widespread inflammation and tissue damage in the affected organs [1]. There are marked disparities in the incidence and prevalence of SLE worldwide, with the highest estimates of incidence and prevalence of 23.2 per 100,000 person-years and 241 per 100,000 people, respectively, in North America [2]. The disease predominantly affects women of childbearing age, with a female to male ratio of 9 to 1. The immunopathogenesis of SLE is multifactorial and complex. Despite the progress in therapeutic options and the improvement in the survival rate, SLE remains an incurable disease [3]. Given the exceedingly rapid proliferation in the number of scholarly articles in recent years [4] and the myriad studies on SLE, it is a challenge to gain a comprehensive overview of the prominent research in SLE.

There are two main approaches in gaining a comprehensive overview of a research field, which can be used in a complementary fashion as a basis for identifying potential knowledge gaps. First, literature reviews, both narrative and systematic, focus on research findings and aim to draw an overall conclusion [5]. Systematic literature reviews differ from narrative literature reviews by having a clearly defined purpose and search approach with explicit inclusion and exclusion criteria [6]. The second approach is through the use of bibliometrics, which is an analytical and mapping method to quantitatively assess the linkages and impact of published articles and citations [7]. This is useful for gaining an overview of the status and trends in a journal [8], research field [9], country [10] or even globally [11]. It also enables the identification of influential authors [12] and publications [13] through the analysis of citation.

Bibliometric analysis, considered as a distinct concept for the first time by Alan Pritchard in 1969 [14], has become a widely applied research methodology in recent years due to the rise in the number of publications for analysis as well as the availability of user-friendly analytical computer programs. Nevertheless, there is a scarcity of bibliometric studies on SLE research to date. One recent study explored the bibliometric profile and collaborative networks of scientific research on systemic lupus erythematosus in Latin America from 1982 to 2018, based on 3843 documents indexed in Scopus. A sustained increase in research in SLE was noted, with Brazil, Mexico, and Argentina generating most publications [15]. Another study investigated a total of 14,053 articles on SLE published in 1627 journals indexed in PubMed from 2002 to 2011. A steady increase in publication in SLE research during the study period was observed. Among the 97 countries and regions identified, the four countries with the highest productivity were the United States, followed by Japan, China, and the United Kingdom [16]. In view of the need to further understanding of the research status of SLE, the aim of the present bibliometric study was to provide an up-to-date overview of SLE research publications between 1971 and 2020.

## 2. Materials and Methods

### 2.1. Source of Bibliometric Data and Search Strategy

The Web of Science (WoS) (Clarivate Analytics, Philadelphia, PA, USA) database was used to identify research articles on SLE. The Science Citation Index Expanded in the WoS Core Collection online database was selected. The following search strategy was used in this study: TS = (systemic lupus erythematosus OR SLE). The search was conducted on 30 January 2021. A timespan of 50 years was set, and therefore only articles published in the years from 1971 to 2020 (based on the WoS field tag PY) were included. Moreover, to ensure proper interpretation of the results, publication language was restricted to English (based on the WoS field tag LA). Only original articles (based on the WoS field tag PT) were included in the bibliometric analysis, and those classified as both articles and other types of documents were excluded.

### 2.2. Bibliometric Analysis

The eligible records retrieved from WoS were first examined for the presence of anomalies using HistCite version 12.03.17 [17]. A bibliometric analysis was performed on the final records using bibliometrix 3.0 (Naples, Italy) (https://www.bibliometrix.org/, accessed on 1 July 2021) [18]. The shiny app Biblioshiny was used to provide a graphical web-interface in the RStudio environment, version 1.3.1093 (https://rstudio.com, accessed on 1 July 2021).

The Journal Impact Factor was obtained from Journal Citation Reports 2018 released by Clarivate Analytics. Moreover, *h*-index, which is defined as the maximum value of *h*, such that the given author or journal has published *h* papers that have each been cited at least *h* times, was used to characterize the scientific output of a journal or author [19]. A treemap chart was generated using the 50 most frequently occurring terms based on “KeyWords Plus”, which are keywords semi-automatically assigned by the editorial team at WoS from the titles of articles. In addition, to illustrate the research hotspots of SLE, keywords co-occurrence was analyzed using VOSviewer software (version 1.6.15) [20].

## 3. Results

In the 50-year period from 1971 to 2020, there were 77,733 English publications indexed in the WOS Core Collection online database. These documents encompassed 46,921 original articles, 15,212 meeting abstracts, 9766 review articles, and 7935 publications of other types. The 46,923 original articles were further examined using HistCite to exclude articles that were indexed with multiple document types (based on the WoS field tag PT). Thus, 1913 articles were removed, which included “article; book chapter” (n = 65), “article; early access” (n = 152); “article; proceedings paper” (n = 1682); and “article; retracted publication” (n = 14). Moreover, 41 articles were excluded because their published year was 2021. Therefore, a total of 1954 articles were removed from the 46,921 original articles, leaving 44,967 articles for the final analysis using bibliometrix (Table 1).

**Figure 1:**
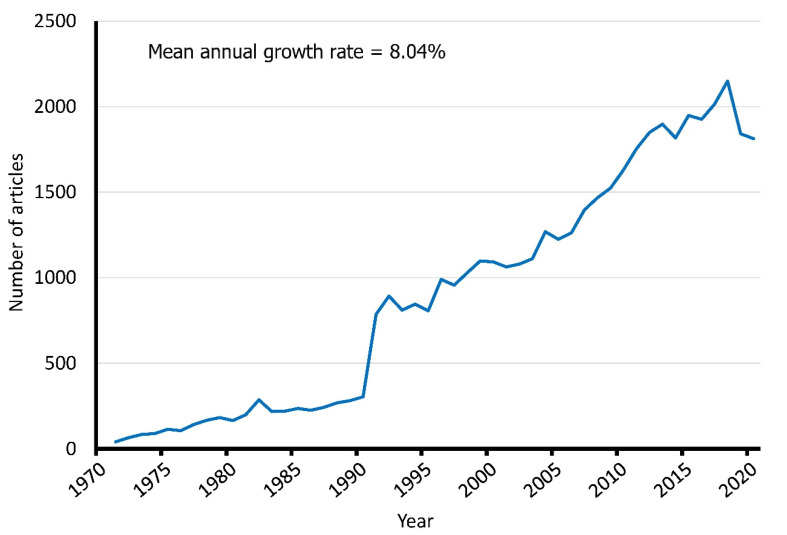
Annual number of original articles on systemic lupus erythematosus from 1971 to 2020.

Figure 1 shows the annual production of original articles on SLE over time. There was a steady increase in annual production except for a steep increase of more than double the number in the year 1991. The peak of production was observed in 2019 with 1841 articles. Overall, a mean annual growth rate of 8.0% was observed.

A total of 148 countries and regions contributed to the articles analyzed in the present study. Table 2 shows the top 10 most productive countries, based on the affiliation of the corresponding author. The United States (n = 11,244, 25.0%) represented the largest share of publications, followed by China (n = 4893, 10.9%), and Japan (n = 3258, 7.2%). The average citation number was the highest in articles with the corresponding author from the United Kingdom, followed by the United States.

In addition, Germany showed the highest ratio of multiple country publication to total publication (29.2%), indicating a high inter-country collaboration within its publications. On the other hand, Japan showed the lowest ratio of 6.4%.

**Figure 2:**
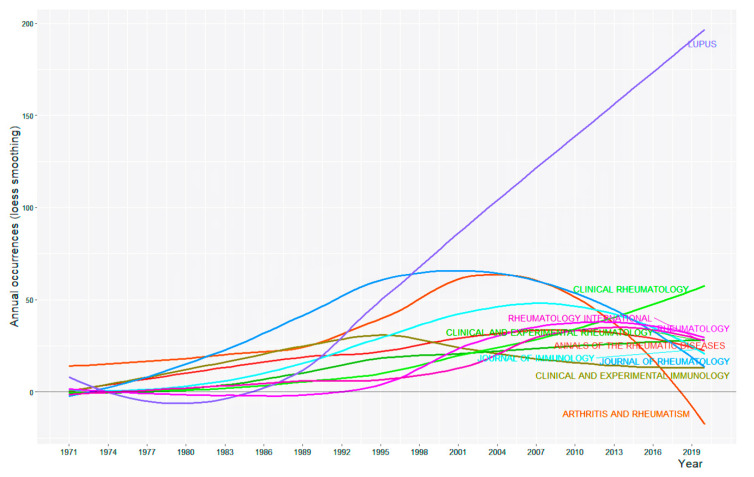
Plot of annual occurrence of articles in the top 10 journals from 1971 to 2020.

The retrieved articles were published in 3435 different journals. A total of 13,125 articles (29.2%) were published in the top 10 journals, which all belonged to the WoS subject category of rheumatology. The journal that had the largest number of articles was *Lupus* (n = 3371). The journal with the highest 2019 Journal Impact Factor (16.102) was *Annals of the Rheumatic Diseases*, whereas that with the highest *h*-index value (167) was *Arthritis and Rheumatism* (Table 3). Figure 2 shows the trend in publication by the top 10 journals over time. A smooth line was plotted using regression analysis based on the loess smoothing technique. The most notable curve is the continuous growth in the number of articles published in *Lupus*. The drop in *Arthritis and Rheumatism,* the official journal of the American College of Rheumatology, beginning in 2011 was because the journal title was discontinued since 2013 [21]. The remainder of the journals showed a relatively steady trend over time.

**Figure 3:**
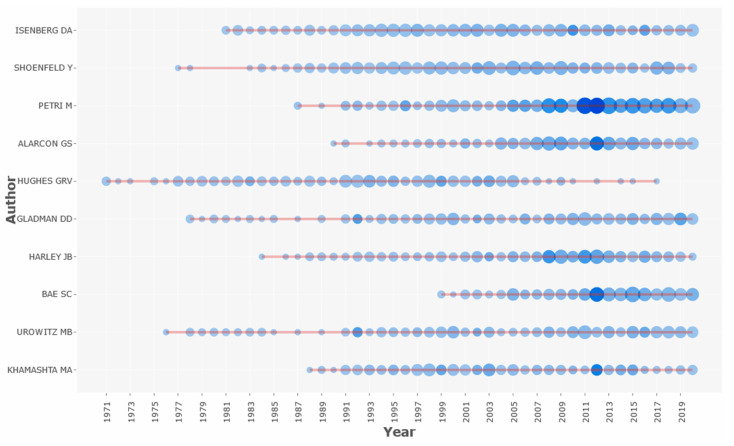
Plot of the top 10 most prolific authors on systemic lupus erythematosus from 1971 to 2020.

A total of 120,834 authors was listed in the 44,967 articles, and among them 1692 (3.8%) were articles with only a single author. Document per author ratio was 0.372, which means, on average, each article had 2.69 authors. Figure 3 shows a plot of the top 10 most prolific authors in SLE research over the study period. The size of the dot represents the number of articles and the intensity of the color represents the total number of citations per year. In terms of the number of articles published during the study period, the top three most prolific authors were Dr. David A. Isenberg and Dr. Yehuda Shoenfeld, each involved in 379 original articles, followed by Dr. Michelle A. Petri, involved in 373 original articles. Conversely, of the 120,834 authors, 81,958 (67.8%) and 17,353 (14.4%) had published only one or two original articles, respectively.

The top 10 leading original articles, based on the number of citations during the study period are shown in Table 4. The top article was by Sakaguchi et al. on immunologic self-tolerance published in 1995 [22]. The second [23] and third articles [24] were both reports on the development of SLE-related measurement scales. All ten articles were published in the 1990s except two. The article published in 2003 demonstrated that anti-double stranded DNA (dsDNA) antibodies preceded the onset of clinical illness in SLE [25]. The other article published in 2012 reported the derivation and validation of Systemic Lupus International Collaborating Clinics classification criteria for SLE, which is still being used to date [26]. Of the 44,967 articles, 2570 (5.7%) received no citations. In addition, the median and mean number of citations per article was 15 and 32.2, respectively.

**Figure 4:**
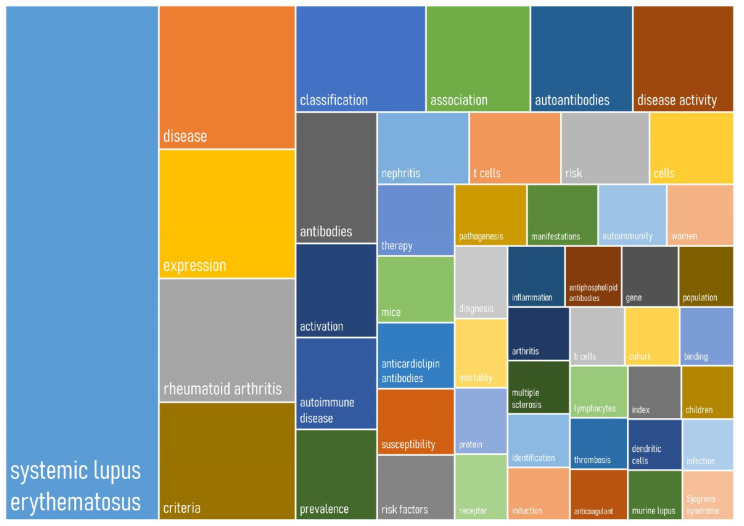
A treemap chart of the 50 most frequently occurring “KeyWords Plus” terms in original articles on systemic lupus erythematosus from 1971 to 2020.

There was a total of 37,321 keywords included in the “Keywords Plus” list. Figure 4 presents a treemap chart of the 50 most frequently occurring terms based on “Keywords Plus”. A larger rectangle area represents a larger proportion of a particular term. Systemic lupus erythematosus, disease, expression, rheumatoid arthritis, and criteria were the top five most prominent terms.

**Figure 5:**
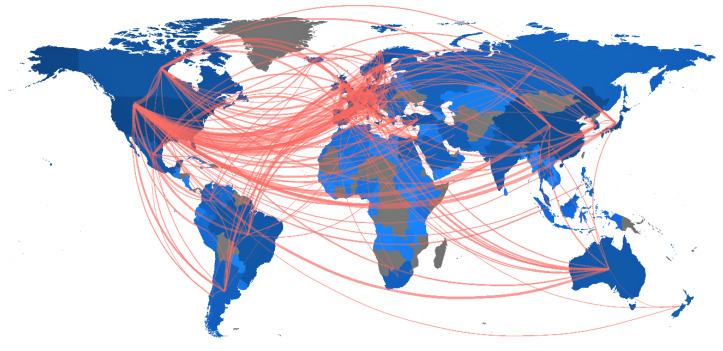
A world map of international research collaboration in original articles on systemic lupus erythematosus from 1971 to 2020.

The international research collaboration in original studies of SLE is shown in Figure 5. The number of published articles is indicated by the intensity of the blue color. The thickness of the red line indicates the strength of the collaboration based on frequency. The top nine strongest collaborations were between the United States and other countries, including the United Kingdom (frequency = 756), China (frequency = 584), Germany (frequency = 427), Sweden (frequency = 384), Japan (frequency = 356), Italy (frequency = 337), Spain (frequency = 315), and France (frequency = 306).

**Figure 6:**
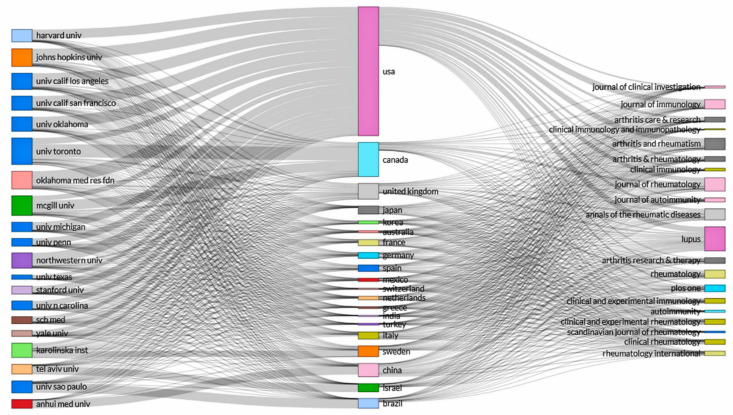
A three-field plot showing the network between institutions (**left**), countries (**middle**), and journals (**right**) of original articles on systemic lupus erythematosus from 1971 to 2020.

A three-field plot based on a Sankey diagram that depicts the connections from countries to institutions and journals is displayed in Figure 6. The height of the rectangle nodes is proportional to the frequency of occurrence of a certain country, institution, or journal within the collaboration network. The width of the lines between the nodes is proportional to the number of connections. The figure shows that the United States (frequency = 48,580) was the country with the most connections, followed by Canada (frequency = 12,868) and the United Kingdom (frequency = 5957). The top contributing institution in the United States was the Oklahoma Medical Research Foundation (4568/48,580 = 9.4%), followed by Johns Hopkins University (4426/48,580 = 9.1%). In Canada, the top contributing institution was the University of Toronto (4950/12,868 = 38.5%), followed by McGill University (3674/12,868 = 28.5%). Regarding the journals on the right, the main contributing countries to *Lupus* was the United States (23.0%) and China (12.2%). In addition, the main contributing countries to *Journal of Rheumatology* was the United States (31.4%) and Canada (19.7%).

**Figure 7:**
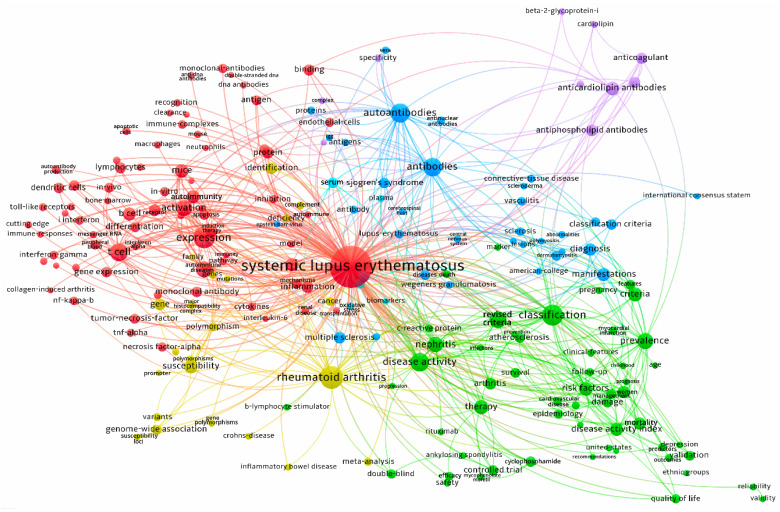
A co-occurrence network of “Keywords Plus” from original articles on systemic lupus erythematosus from 2001 to 2020.

The co-occurrence network of “Keywords Plus” terms is shown in Figure 7. The purpose of co-occurrence analysis is to access the relatedness of items based on the number of documents in which they occur together. There was a total of 37,322 unique terms recorded in the articles. To achieve adequate readability, only the top 200 terms were included to generate the co-occurrence network. The size of the circle reflects the number of articles in which the term occurs. The proximity of two linked terms presents the relatedness of the terms based on their number of co-occurrences. A total of five interconnected clusters were observed, including a main cluster of systemic lupus erythematosus (red), classification (green), rheumatoid arthritis (yellow), autoantibodies and antibodies (blue), and a small cluster of anticardiolipin antibodies (purple).

## 4. Discussion

In this bibliometric study, the literature on SLE research published in the WoS database from 1971 to 2020 was analyzed using bibliometrix 3.0 and VOSviewer. There was a total of 44,967 original articles on SLE published during the past 50 years. Overall, there is a steady increase in the number of original articles in SLE with a mean annual growth rate of 8.0%. With an 8% growth rate, it would take only 8.75 years for the number of articles to double. While increased research funding over time could be a reason for this growth [27], the pressure-to-publish culture among academia in recent times [28,29] might also have played a role. In addition, the steep rise from 1990 to 1991 could be attributed to a change in the abstracting algorithm in WoS in 1990, where KeyWords Plus indexing was introduced in 1990 [30]. Such a phenomenon could also be observed in other bibliometric analyses based on the WoS database [31,32,33].

Of the 148 countries and regions analyzed, the United States was the leading country, contributing 25% of the total number of publications. Many factors could contribute to the disparity in publication output among different nations. A correlational study based on 5275 original articles published in the five highest ranked general medical journals between 1996 and 2001 suggested that research funding levels and English fluency were significantly associated with publication output [34]. Another study on 1107 articles in three major biomedical journals found that articles with corresponding authors living in the same country as that of the publishing journal were twice as likely as others to be accepted in *British Medical Journal*, *The Lancet*, and *Annals of Internal Medicine* [35]. A recent study based on country-level data showed that Internet penetration of a country was significantly associated with the quantity of research output [36]. In the present study, the top 10 most productive countries were the same as those reported by a bibliometric study of SLE research between 2002 to 2011 based on all articles published in journals indexed by the PubMed database, with only minor differences in sequence [16].

There is a global trend in science towards international collaboration [37,38]. Our world map of international research demonstrated that the United States had the highest collaboration network, particularly with the United Kingdom. The three-field plot showed that the Oklahoma Medical Research Foundation and the Johns Hopkins University contributed 18.5% of all publications from the United States. Nevertheless, from the point of view of the number of citations, publications from the United Kingdom showed a slightly higher average article citation of 45.3 compared with the 45.0 from the United States.

A total of 3435 different journals have published original articles on SLE. All of them were listed under the subject category of rheumatology in WoS. In terms of the number of publications, *Lupus* published the most articles with a contribution of 7.5%. The top 10 journals identified in the present study were similar to those reported in the bibliometric study of SLE research between 2002 to 2011 by Li et al. [16], with only two exceptions. First, *Clinical and Experimental Immunology* ranked 7th in the present study, but 14th in Li et al. study. Second, *Autoimmunity Reviews*, ranked 10th in Li et al. study, was not in the list of our study. This journal was established relatively recently in 2002 and had an impact factor of 7.77 in 2019. It published only structured reviews on topics in autoimmunity, and articles are principally solicited by the Editors-in-Chief.

The most cited original article by Sakaguchi et al. published in 1995 was a seminal paper that reignited interest in regulatory T cells by demonstrating that a small CD4^+^CD25^+^ T cell population had suppressive activity [22]. The second and third most cited articles were both reports of development of SLE-related measurement scales. Bombardier et al. reported the derivation of a disease activity index for patients with SLE, the SLEDAI, which was based on the consensus rating of 574 patient profiles by a group of rheumatologists [23]. Krupp et al. devised a nine-item fatigue severity scale for application to patients with multiple sclerosis and systemic lupus erythematosus. The scale was well adopted in studies of these disorders as well as a variety of other disorders [24]. While we have identified the top 10 most cited original articles, many other landmark publications existed, especially in clinical trials, such as those on belimumab [39,40] and rituximab [41]. A separate bibliometric analysis with a search limited to only clinical studies will be needed to explore this aspect.

According to the results of the co-occurrence network analysis, five highly connected clusters were observed. The main cluster (red) was SLE connected to terms related to gene expression, activation, b cells, and t cells. The next highly connected cluster (green) was concerned mostly with clinical aspects of SLE, including disease classification, diagnostic criteria, disease activity, epidemiology, study methodology, and outcomes. The third cluster (blue) linked studies on antibodies and autoantibodies with various autoimmune diseases. As SLE is characterized by the formation and deposition of autoantibodies directed against nuclear self-antigens and circulating immune complexes, it is not surprising to observe a large cluster of keywords surrounding the topic. The fourth cluster (yellow) is concerned with studies in identifying risk loci shared between SLE and rheumatoid arthritis as well as other autoimmune diseases. The appearance of genome-wide association studies (GWAS) has marked the start of a new era of genetic research on SLE. Before 2007, there were only nine established SLE-susceptibility loci identified, but the number has increased to more than 25 in just two years by 2009 with the use of GWAS [42]. The number has further grown to over 100 by 2019 [43]. The fifth cluster (purple) showed the connection between SLE with antiphospholipid antibodies, anticardiolipin antibodies, and beta-2 glycoprotein 1. Approximately 30%–40% of patients with SLE possess antiphospholipid antibodies, and anticardiolipin antibodies and anti-beta-2 glycoprotein 1 are commonly studied entities. Since there is an increased risk of thrombosis in individuals with anticardiolipin antibodies, it is also not surprising that the term anticoagulant was closely linked to it [44]. Overall, the identified clusters are consistent with what is known about SLE research.

This study has a number of limitations worth mentioning. First, the articles analyzed in this study were retrieved from the WoS. While it is well-known that there are strengths and weaknesses in various databases [45], we chose WoS over PubMed because the latter database does not store citation metadata, but citations are a vital part of research output indicators. The use of WoS also complemented the two other bibliometric studies on SLE, which used Scopus [15] and PubMed [16]. Second, as in all bibliometric studies, a potential length time-effect bias exists, which puts the more recent articles at a disadvantage in receiving citations. Third, misclassification of document type for research letters is possible. Research letters are classified as either “letters” or “articles” on a paper-to-paper basis based on a number of attributes, such as the placement in a journal, author instructions, and the presence of an abstract or references [46].

## 5. Conclusions

The present study offered an overview of the status of SLE research production in the past 50 years, as reflected by original articles indexed by the WoS Core Collection database. Our findings identified prominent countries, institutions, journals, original articles, and authors to indicate the most influential research channels. Despite advances in the understanding of its etiology, pathogenesis, and disease management, SLE is still associated with significant comorbidity and impact on health-related quality of life. It is hoped that this bibliometric analysis will provide useful information for determining research and publication strategies in future investigations of SLE.

## Figures and Tables

**Table 1:** Distribution of document types on systemic lupus erythematosus research published between 1971 and 2020 (N = 77,733).

Document Type	N (%)	
	Raw Data	Data After Data Cleansing
Article	46,921 (60.4)	44,967
Meeting abstract	15,212 (19.6)	
Review	9766 (12.6)	
Letter	2886 (3.7)	
Editorial material	1730 (2.2)	
Proceedings paper	1677 (2.2)	
Note	880 (1.1)	
Other	762 (1.0)	

Only “article” was included in the bibliometric analysis of the present study. The “other” document type included correction (n = 235), early access (n = 218), book chapter (n = 191), discussion (n = 42), news item (n = 35), retraction (n = 21), reprint (n = 8), biographical item (n = 7), abstract of published item (n = 2), book review (n = 2), and database review (n = 1). The total does not add up to 100% because an article could be classified into more than one document type.

**Table 2:** The top 10 most productive countries, based on the affiliation of the corresponding author of original articles on systemic lupus erythematosus from 1971 to 2020.

Rank	Country	Number of Articles (%)	Average Article Citations	MCP/Total Ratio (%)
1	The United States	11,244 (25.0)	45.0	16.4
2	China	4893 (10.9)	15.4	11.5
3	Japan	3258 (7.2)	21.4	6.4
4	The United Kingdom	2163 (4.8)	45.3	27.2
5	Italy	1624 (3.6)	29.1	20.2
6	Germany	1453 (3.2)	36.9	29.2
7	France	1176 (2.6)	36.2	21.1
8	Canada	1168 (2.6)	40.8	29.0
9	Spain	1115 (2.5)	31.6	25.1
10	Brazil	1030 (2.3)	14.3	10.9

MCP: multiple country publication.

**Table 3:** The top 10 journals based on the number of original articles on systemic lupus erythematosus.

Rank	Journal	Number of Articles (%)	Journal Impact Factor ^1^ (Quartile)	*h-*Index ^2^
1	*Lupus*	3371 (7.5)	2.251 (1)	87
2	*Journal of Rheumatology*	1893 (4.2)	3.350 (2)	104
3	*Arthritis and Rheumatism* ^3^	1616 (3.6)	8.955 (1)	167
4	*Journal of Immunology*	1238 (2.8)	4.886 (2)	124
5	*Annals of the Rheumatic Diseases*	1054 (2.3)	16.102 (1)	107
6	*Clinical Rheumatology*	874 (1.9)	2.394 (3)	44
7	*Clinical and Experimental Immunology*	865 (1.9)	3.532 (2)	72
8	*Rheumatology*	743 (1.6)	5.606 (1)	79
9	*Clinical and Experimental Rheumatology*	737 (1.6)	3.319 (2)	54
10	*Rheumatology International*	734 (1.6)	1.984 (3)	38

^1^ Journal Impact Factors were obtained from the Journal Citation Reports, calculated based on the citations in 2019 divided by the total number of citable items in 2017 and 2018. ^2^ The *h*-index, also known as the Hirsch index, is defined as the maximum value of *h* such that the given journal has published *h* papers that have each been cited at least *h* times. ^3^ The title discontinued in 2013 and therefore the 2015 Journal Impact Factor was used.

**Table 4:** The top 10 original articles based on the number of citations in systemic lupus erythematosus research between 1971 and 2020.

Rank	First Author (No. of Total Authors)	Title	Journal	Year of Publication	Total Citations	Total Citations/Year
1	Sakaguchi S (5)	Immunologic self-tolerance maintained by activated T cells expressing IL-2 receptor alpha-chains (CD25). Breakdown of a single mechanism of self-tolerance causes various autoimmune diseases	*Journal of Immunology*	1995	4415	163.5
2	Bombardier C (5)	Derivation of the SLEDAI. A disease activity index for lupus patients. The Committee on Prognosis Studies in SLE	*Arthritis and Rheumatism*	1992	3610	120.3
3	Krupp LB (4)	The fatigue severity scale. Application to patients with multiple sclerosis and systemic lupus erythematosus	*Archives of Neurology*	1989	3147	95.4
4	Kreig AM (8)	CpG motifs in bacterial DNA trigger direct B-cell activation	*Nature*	1995	2783	103.1
5	Watanabe-Fukunaga R (5)	Lymphoproliferation disorder in mice explained by defects in Fas antigen that mediates apoptosis	*Nature*	1992	2531	84.4
6	Wilson WA (12)	International consensus statement on preliminary classification criteria for definite antiphospholipid syndrome: report of an international workshop	*Arthritis and Rheumatism*	1999	1943	84.5
7	Wilson AG (5)	Effects of a polymorphism in the human tumor necrosis factor alpha promoter on transcriptional activation	*Proceedings of the National Academy of Sciences of the United States of America*	1997	1879	75.2
8	Petri M (52)	Derivation and validation of the Systemic Lupus International Collaborating Clinics classification criteria for systemic lupus erythematosus	*Arthritis and Rheumatism*	2012	1793	179.3
9	Gladman D (22)	The development and initial validation of the Systemic Lupus International Collaborating Clinics/American College of Rheumatology damage index for systemic lupus erythematosus	*Arthritis and Rheumatism*	1996	1566	60.2
10	Arbuckle MR (7)	Development of autoantibodies before the clinical onset of systemic lupus erythematosus	*New England Journal of Medicine*	2003	1505	79.2

## Data Availability

The data that support the findings of this study are available from the corresponding author upon reasonable request.
